# Dismantling of Waste Printed Circuit Boards with the Simultaneous Recovery of Copper: Experimental Study and Process Modeling

**DOI:** 10.3390/ma14185186

**Published:** 2021-09-09

**Authors:** Szabolcs Fogarasi, Árpád Imre-Lucaci, Florica Imre-Lucaci

**Affiliations:** 1 Department of Chemical Engineering, Faculty of Chemistry and Chemical Engineering, Babeş-Bolyai University, 11 Arany Janos Street, 400028 Cluj-Napoca, Romania; szabolcs.fogarasi@ubbcluj.ro; 2 Interdisciplinary Research Institute on Bio-Nano-Sciences, Babeş-Bolyai University, 42 Treboniu Laurian Street, 400271 Cluj-Napoca, Romania

**Keywords:** WPCBs dismantling, metals dissolution, copper recovery, economic assessment, process modeling and simulation

## Abstract

The study was carried out with the aim to demonstrate the applicability of a combined chemical–electrochemical process for the dismantling of waste printed circuit boards (WPCBs) created from different types of electronic equipment. The concept implies a simple and less polluting process that allows the chemical dismantling of WPCBs with the simultaneous recovery of copper from the leaching solution and the regeneration of the leaching agent. In order to assess the performance of the dismantling process, various tests were performed on different types of WPCBs using the 0.3 M FeCl_3_ in 0.5 M HCl leaching system. The experimental results show that, through the leaching process, the electronic components (EC) together with other fractions can be efficiently dismounted from the surface of WPCBs, with the parallel electrowinning of copper from the copper rich leaching solution. In addition, the process was scaled up for the dismantling of 100 kg/h WPCBs and modeled and simulated using process flow modelling software ChemCAD in order to assess the impact of all steps and equipment on the technical and environmental performance of the overall process. According to the results, the dismantling of 1 kg of WPCBs requires a total energy of 0.48 kWh, and the process can be performed with an overall low environmental impact based on the obtained general environmental indexes (GEIs) values.

## 1. Introduction

According to the literature data, in the period 2016–2030, the quantity of waste electrical and electronic equipment (WEEEs) will increase at world level from 44.7 million tonnes to 110 million tonnes [1,2,3,4]. Thus, increasing the quantity of WEEEs requires the urgent development of environmentally friendly and low cost recycling technologies [5]. Waste printed circuit boards (WPCBs) represents an important part of WEEEs (3–5 wt.%) with more than 30% metals (of which ~30% copper) and 70% non-metals [6,7,8]. For this reason, the recycling of WPCBs can constitute a potential secondary source of raw materials for different industrial sectors [9], contributing to preservation of natural resources [10,11,12]. With this regard, a lot of research has been done that has established different techniques for WPCBs recycling based on physico-mechanical [13,14], pyrometallurgical [15], pyrolytic [11,16,17], and hydrometallurgical approaches [18,19]. One of the major issues that needs to be solved regarding the recycling of WPCBs is related to its dismantling into different material fractions that can be further processed into value added products [20,21]. Some studies so far conducted to WPCBs dismantling involve the thermal processing of WPCBs, but this operation is not eco-friendly because it occurs with the release of toxic gases [22], high energy consumption, and the burning of components that could be reused [23,24]. On the other hand, hydrometallurgical recovery techniques of valuable materials from WPCBs are achieved by grinding followed by acid or basic leaching of base metals from the obtained powder with the generation of other unusable or toxic residues; at the same time, they are non-selective processes [25,26,27]. Typically, the approach does not allow the full recovery of metallic copper, which is the main base metal of the EC found on the motherboards. Considering that traditional approaches are highly energy-demanding and environmentally dangerous [28,29], our research group has been carrying out the development of chemical and electrochemical processes for the recovery of metals from WPCBs, which allow the oxidative dissolution of metals with the simultaneous electrochemical regeneration of the oxidant, leading to the minimization of several drawbacks [30,31,32,33]. It was found that the metallic parts that hold together the different parts of WPCBs can be dissolved in the combined chemical–electrochemical processes, leading to the disassembly of WPCBs into different material fractions, such as plastics, printed circuit boards without EC, chips and small EC, and sludge [31,33,34,35]. Considering the success achieved with particular types of WPCBs [31], in this work, it is our intention to prove the applicability of this method for the dismantling of other types of WPCBs simultaneously with the electrochemical regeneration of the leaching agent and the partial electrodeposition of dissolved copper. In addition, to evaluate the contribution of all stages and equipment to the technical–environmental performance of the process, besides the ones used in the experimental studies, the process was extended, modeled, and simulated using ChemCAD process flow modelling software for a higher productivity.

In order to accomplish the abovementioned aims, the following sections are discussed after the materials and methods section: (i) *Theoretical background for the dissolution of metals* to better understand the processes that occur in the case of WPCBs disassembly; (ii) *experimental dismantling process of WPCBs* meant to provide the necessary background for the scale up of the process; (iii) *the scaled-up dismantling process* in order to evaluate the contribution of all steps and equipment to the performance of the overall process and to provide the necessary data for the environmental assessment; (iv) *environmental assessment of the scaled-up dismantling process* to assess the environmental impact of the process in the early phase of development using the Biwer–Heinzle method. 

## 2. Materials and Methods

The WPCB dismantling experiments were performed using four types of motherboards, which differ in terms of technical details (Table 1) and metal content (Table 2) due to the different periods of production. For each case, before inserting the WPCBs into the rotating drum of the chemical reactor (CR), large pieces of aluminum and stainless steel were removed from the surface of the WPCBs, because they can be easier recycled this way. Additionally, it is not justified, due to the high power consumption, to bring the metals from these parts into the solution. Next, the WPCBs were cut into 40–100 cm^2^ pieces to fit into the rotating drum. 

The experimental setup used for the dismantling of the WPCBs samples consisted of two reactors connected in series, including a 2L CR with perforated rotating drum and a 3L divided electrochemical reactor (ER) by a ceramic separator. In all experiments, 5L of 0.3 M FeCl_3_ in 0.5 M HCl solution was recirculated between the two reactors using two Medorex TC200 pumps (Medorex, Nörten-Hardenberg, Germany). The solution was evacuated at the bottom of the CR and supplied at the bottom of the cathode compartment of the ER. From the top of the cathode compartment, the solution was transported to the bottom of the anode compartment, and with the help of the second pump, the solution was pumped from the top of the anode compartment back into the CR. In consequence, a cross flow of electrolyte between the two reactors is achieved. The rectangular cathode and anode were made of copper and graphite, respectively, each with an area of 570 cm^2^. Two Ag/AgCl/KCl_sat_ reference electrodes were used to measure the cathodic and anodic potentials. The experiments were carried out in the optimal operating conditions identified for the combined chemical-electrochemical processes in previous studies [31,32,36]: drum rotation speed (30 rpm), solid/liquid ratio (1/8), constant current density 4 mA/cm^2^, initial electrolyte composition 0.3 M FeCl_3_ in 0.5 M HCl, and a flow rate of 400 mL/min. An atomic absorption spectrometer was used to determine the metal content and different material fractions of the solutions, while the surface morphology and chemical composition of the cathodic deposits were characterized with a scanning electron microscope (SEM, Thermo Fisher Scientific, Waltham, MA, USA) equipped with an energy dispersive X-ray spectrometer (SEM/EDAX, FEI QUANTA 3D).

## 3. Results and Discussions

### 3.1. Theoretical Background for the Dissolution of Metals 

To better understand the processes that occur in the case of WPCB disassembly, through the dissolution of metals with Fe^3+^ in the presence of Cl^−^, the standard apparent potentials (*E*’) were calculated for the potentially active reactions (PAR) based on the standard normal potentials (*E*^0^) and the thermodynamic equilibrium constants for the formation of complexes [37,38,39]. In order to calculate the *E*, it is necessary to identify the chemical species present in the solution, with respect to the equilibriums in which they are involved. In the case of copper, the species involved in the process were considered on the basis of the Pourbaix diagram for the copper–chlorine–water system at 25 °C at a total Cl^−^ concentration of 1.5 M [40].

In the case of the other metals, the species involved in the chemical process have been identified from the literature based on their stability in the reaction environment [37,38,41,42]. The *E*’ values (Table 3) were obtained (Equation (1)) based on the Nernst equation applied for redox reactions, which involves the complexation of the oxidized form of the redox couple [21].

(1) $$E^{\prime }=E^{0\;}–\;\frac{0.059}{n}logK_{f_{ox}}$$

where *E*^0^ is the standard normal potential, *n* is the number of electrons changed, and $K_{f_{ox}}$ is the thermodynamic equilibrium constant for the formation of complexes with the oxidized form of the redox couple.

As was expected, the standard apparent potential decreases when the complexing agent employs the oxidized form, and the potential shift will be greater as the stability of the complexes is higher. Therefore, *E’* differs the most from *E*^0^ in the case of Au, Ag, and Cu(I), for which the thermodynamic equilibrium constants for the formation of chloro-complexes are the highest. In the case of the other metals, where the complexation equilibrium is less shifted to the formation of chloro-complexes, the redox potential varies insignificantly under the experimental conditions. Additionally, from the E’ values calculated for the PAR, it can be seen that Fe^3+^ is an efficient oxidant in the dissolution of metals, with the exception of gold. 

It is also important to note that the dissolution processes lead to chloride complexes of Cu, Sn, Pb, Fe, in which the metals may have different oxidation forms. As a result, the *E’* values (Table 4) for these redox couples, in which both forms are involved in complexation processes, was calculated by the following equation:

(2) $$E^{\prime }=E^{0\;}+\frac{0.059}{n}log\frac{K_{f_{ox}}}{K_{f_{red}}}.\;$$

As can be seen from Table 4, the existence of significant differences between the E’ values of these redox couples implies a state of un-equilibrium in these working conditions. The evolution of the system towards equilibrium determines the change in the concentration of the electroactive species, especially in the case of metals that may have different oxidation forms. The most important redox reactions leading to the installation of redox equilibrium are those in which Fe^3+^ oxidizes Cu^+^ to Cu^2+^ and Sn^2+^ to Sn^4+^. From the strong positive value of E’ for the couple ${\mathrm{PbCl}}_{6}^{2-}/{\mathrm{PbCl}}_{4}^{2-}$, it is concluded that Pb is more stable in the reduced form ${\mathrm{PbCl}}_{4}^{2-}$.

Therefore, from the oxidation reaction of Pb by Fe^3+^, only Pb^2+^ is formed without its subsequent oxidation to Pb^4+^. The above conclusions are in agreement with the values of redox equilibrium constants (K_r_) for the dissolution reactions of the metals from the WPCBs samples, calculated on the basis of Equation (4) using the E’ values from Table 3 and Table 4.

For a redox reaction in the general form:

(3) $${m\;\mathrm{ox}}_{1}+{\;p\;\mathrm{red}}_{2}⇌{m\;\mathrm{red}}_{1}+{\;p\;\mathrm{ox}}_{2}$$

The equilibrium constant is defined as follows [32]:

(4) $$K_{r}=10^{\frac{\mathrm{mp}\left({E\;}_{1}^{\prime }-{E\;}_{2}^{\prime }\right)}{0.059}}$$

where mp—number of electrons transferred between the redox couples. 

The K_r_ values increase (the equilibrium will be shifted to the right) with the number of electrons transferred between the redox couples and greater the difference between the values of *E’* for the two systems. The values of the redox equilibrium constants, calculated according to Equation (4), are presented in Table 5.

From the values of redox equilibrium constants, Table 5, it is observed that, for all metals except gold, redox reactions are strongly displaced towards their dissolution with the formation of chloro-complexes. Based on the redox equilibrium constant values from Table 5, it can be assumed that the dissolution rate of metals will have following order: Zn > Sn > Fe > Ni > Pb > Cu > Ag. Tin is located after Zn, although from the E’ value from Table 3, Sn should be between Pb and Cu. This can be explained by the fact that the redox equilibrium constant depends both on the potential difference between the redox couples and on the number of electrons transferred. Considering that Sn changes 4e^−^ while the other metals change only 2e^−^, the redox equilibrium constant is much higher (1.42 × 10^49^), which is assumed to promote its dissolution.

### 3.2. Experimental Dismantling Process of WPCBs

The results presented in Table 6 prove that the combined chemical–electrochemical process leads to the complete dismantling of the WPCBs samples, leading to different material fractions. It can be observed that the obtained fractions can be easily separated due to the major differences in physical characteristic, such as size and density. 

The obtained material fractions still have in their composition undissolved metals, considering that, in the dismantling process, only the metals, which were accessible to the leaching solution, from the surface of the WPCBs were dissolved. To determine their metallic composition, they were grounded to a fine powder and treated with aqua regia to determine the metallic composition, which is presented in Table 7. It can be noticed that, for all the samples, the plastic fractions do not contain metals, which means that all the pins were dissolved. Similarly, the boards contain only copper in a relatively high concentration, and this material can be further processed for high purity copper recovery. As for the other two solid fractions, all the metals are present with the remark that they have high Au and Ag concentration, especially in the case of the obtained sludge. Additionally, Table 7 indicates that almost 70% of the metals present in the initial samples were dissolved during the dismantling process, and the major component is Cu, which represents, on average, 50% of the dissolved metals. 

**Figure 1:**
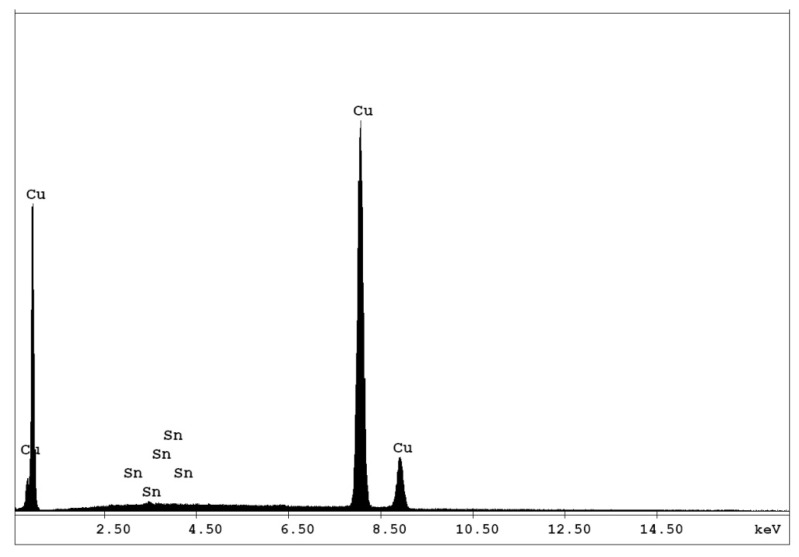
EDAX spectrum of the copper deposit.

This is the main reason why the leaching solution is suitable for high purity copper production simultaneously with the regeneration of the leaching agent and dismantling of the WPCB samples. Considering that the cathodic deposits were obtained at the same current density and flow rate and a similar solution composition, they showed very similar, almost identical, morphological properties and elementary composition. According to the composition analysis, in all the experimental studies, the cathodic deposit contains more than 99.95 wt.% copper, the only impurity being Sn. The elementary analysis results presented in Figure 1 confirm that the developed process leads to the recovery of a high purity copper deposit. It is also important to note that the obtained copper deposits were compact, which is sustained by the characterization made by SEM. 

**Figure 2:**
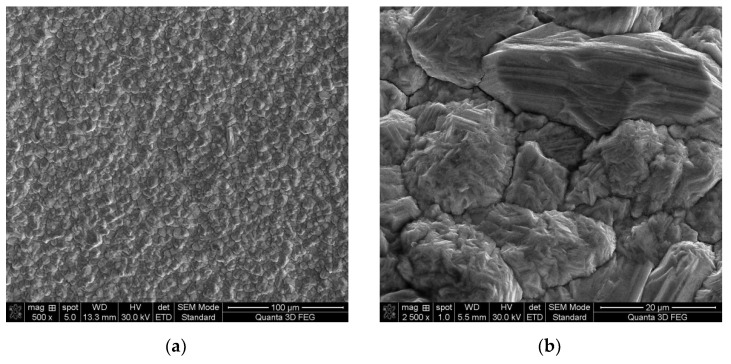
SEM images for the copper deposit at magnitude: ×500 (**a**); ×2500 (**b**).

In addition, the SEM images from Figure 2 show that the copper deposit presents rougher surfaces with larger nuclei and pyramidal growth, which is characteristic for copper deposition from chloride solutions in accordance with the literature data [43]. 

### 3.3. The Scaled-Up Dismantling Process

The experimental results obtained for the dismantling of the four types of WPCBs were used to design a conceptual recycling plant at higher scale of production for the dismantling of 100 kg/h of WPCBs. It was assumed that the WPCBs fed into the recycling plant have the metallic composition of Table 8, which presents an average composition calculated based on the data from Table 2.

The dismantling process was modeled and simulated using process flow modeling software ChemCAD 7.1.5 in order to evaluate the contribution of all steps and equipment to the performance of the overall process and to provide the necessary data for the environmental assessment. Chemical and phase equilibrium were assumed based on Gibbs free energy minimization model. Other or complementary input data and assumptions used for modelling and simulation of the dismantling process are presented in Table 9.

**Figure 3:**
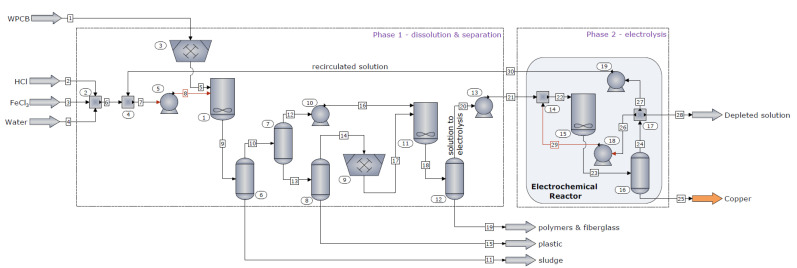
Recycling plant process flow diagram.

Figure 3 shows that the recycling plant designed for the dismantling of WPCBs with the simultaneous recovery of copper includes two main subsystems: Phase 1 for the dissolution of the accessible metallic parts from the surface of WPCBs along with the separation of the obtained material fractions and Phase 2, where copper is electrodeposited and the leaching solution is regenerated. In addition, Phase 1 deals with the processing of the chips, small EC, and boards, considering that these fractions have high copper content and low concentration in other metals. In the model, these material fractions are ground to a fine powder in order to enhance the dissolution reaction; then, the obtained material is contacted in a second chemical reactor with the leaching solution generating two streams: (i) the electrolyte solution used for copper and leaching agent production and (ii) polymers and fiberglass that exit the system as byproduct. According to Figure 3, the solution obtained in the ER is only partially recirculated into the process, due to the fact that it contains other metals besides copper that need to be extracted, after which it can be reused in the dissolution step. This is the reason why, in this model, new reagents are feed into the system, which, in a complete recovery plant, would be significantly, or possibly entirely, reduced. 

The overall mass balance (Table 10) of the scaled-up dismantling process indicates that the WPCBs were completely dismantled and processed into different materials fractions. Based on the initial amount of copper feed into the system with the WPCBs and the amount of obtained copper deposit, it is evident that more 90% of the copper is extracted during the dismantling process. As was expected, the organic matter, consisting of plastic and epoxy resin, represents the most important solid fraction obtained in the dismantling process, followed by fiber glass. It can also be seen that the amount of solution evacuated from the system is slightly higher than at the inlet due to the presents of the dissolved metals. 

In accordance with the mass balance data (Table 10), equipment types, and operating conditions, the overall energy balance of the scaled-up dismantling process was established (Table 11). The results indicate that the highest energy consumption is associated with the electrochemical process followed by the separation of different material fractions during the dismantling process. Combining the mass and energy balance data, it was determined that the dismantling of 1 kg of WPCBs requires 0.48 kWh, while the total specific energy consumption for copper production is 2.59 kWh/kg. It is also important to note that the overall energy balance data reveals that the process generates 47% more energy that it consumes. However, the potential usability of the generated energy is discussible, considering that it is low grad heat generated during the dissolution processes in comparison to the consumed electrical power. However, if the energy would be valorized for heating purposes, then the process would be useful for energy production in parallel with the dismantling of WPCBs and recovery of copper. 

### 3.4. Environmental Assessment of the Scaled-Up Dismantling Process

**Figure 4:**
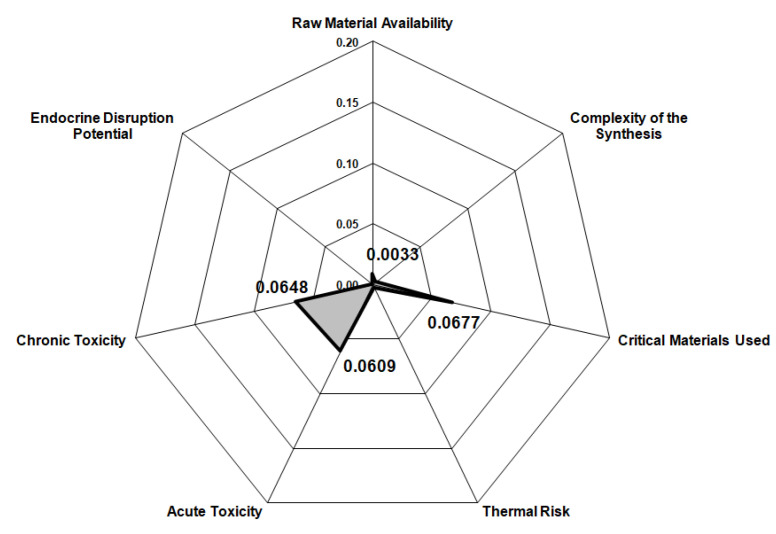
Environmental impact assessment for input streams.

**Figure 5:**
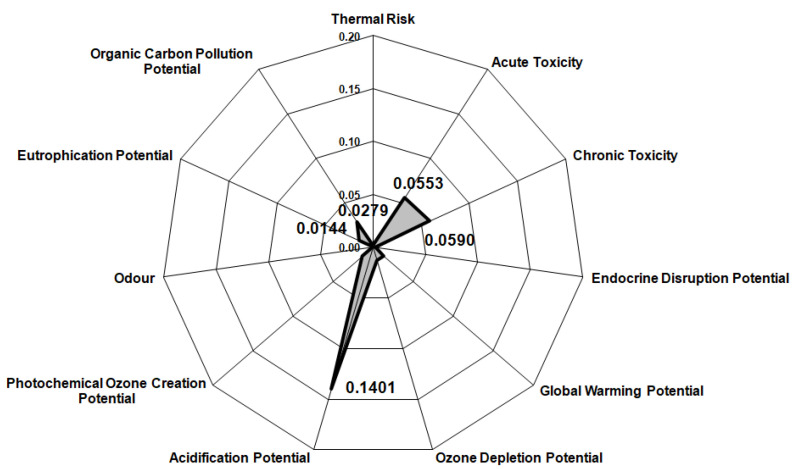
Environmental impact assessment for output streams.

Considering that the process presented in the current study is in the early phase of development, the Biwer–Heinzle method was applied for its environmental impact assessment. According to the methodology described in the literature [30,44] the environmental factors were determined for six impact groups in the case of the input materials (Figure 4) and 11 impact groups in the case of the output materials (Figure 5) by allocating each of the materials streams to a class of toxicity (A = 1—highly toxic substances, B = 0.3—less toxic substances, C = 0—nontoxic substances). The general effect indices (GEIs) were also calculated by dividing the sum of environmental indices to the total mass indices obtained from the mass balance data (Table 9) of the dismantling process.

It was found that the category “critical materials used” has the highest value due to the large amount of FeCl_3_ feed into the process. Additionally, FeCl_3_ is the main reason for the high chronic and acute toxicity of the input material stream. Among the other input streams, HCl and copper have the most important contributions to the results shown in Figure 4. The reason that copper surpasses other toxic components in the WPCBs, such as lead, is due to the fact that, even if it is less toxic than other components, it has the highest concentration. Similarly, for the output materials, chronic and acute toxicity remain important categories, but they are preceded by the “acidification potential” due to the depleted solution. Nevertheless, the GEIs values calculated for the input materials (0.064) is lower than for the output materials (0.039) indicating that the process diminishes the environmental impact of the WPCBs. Moreover, considering that both values are close to the minimum possible (0), it can be concluded that the dismantling process has an overall low environmental impact. 

## 4. Conclusions

The obtained results prove that the combined chemical–electrochemical process can be applied efficiently for the dismantling of different types of WPCBs with the parallel recovery of copper and regeneration of the leaching agent. As it was expected, in accordance with the redox equilibrium constants, all the metals, with the exception of gold, were dissolved during the leaching process. The experimental results demonstrate that the high purity (>99.95%) copper deposit can be produced with high current efficiency (72.69%) and low specific energy consumption (1.59 kWh/kg Cu). It is also important to note that the obtained copper deposits were compact and presented rough surfaces with larger nuclei and pyramidal growth, which is characteristic for copper deposition from chloride solutions. Modeling and simulating the conceptual recycling plant led to the conclusion that more than 90% of the copper can be extracted during the dismantling process by introducing a complementary step for the mechanical pretreatment of the boards, EC, and chips. It was also found that the dismantling of 1 kg of WPCBs requires 0.48 kWh, while the total specific energy consumption for copper production is 2.59 kWh/kg if the energy consumption of all process steps is considered. The overall energy balance revealed the possibility to use the process for a combined recycling–heating purpose, considering that it generates 47% more heat than the electrical power consumed. Based on the results of the environmental impact assessment, it can be concluded that the dismantling process can be performed not just with high technical performance but with low environmental impact as well. Still, further work is recommended to improve the technical performances and to assess the economic potential of the developed conceptual recycling plant. 

## Figures and Tables

**Table 1:** WPCBs samples used in the experimental studies.

Motherboard 1	Motherboard 2	Motherboard 3	Motherboard 4
Type: ATI Radeon Xpress 200 (2004–>) Processor (Socket 478): Pentium 4	Type: MSI P54C TR4 (1996–>) Processor (Socket 7): Pentium; AMD K5; Cyrix 6 × 86	Type: Gigabyte GA-BX2000 (1999–>) Processor (Slot 1): Pentium II; Pentium III	Type: Intel Acorp 6VIA/ZX85 (2000–>) Processor (Socket 370): Pentium III
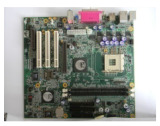	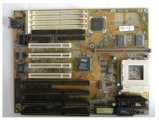	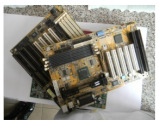	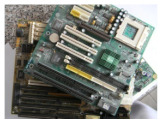

**Table 2:** Concentration (wt.%) of the most important metals in the WPCBs samples.

Sample	Mass, g	Cu	Au	Ag	Sn	Pb	Ni	Zn	Fe
Motherboard 1	459.14	18.29	0.011	0.018	3.92	2.31	1.11	1.71	2.81
Motherboard 2	488.45	19.6	0.009	0.015	4.1	2.16	1.27	1.92	3.01
Motherboard 3	444.06	22.1	0.013	0.02	3.51	2.59	1.06	1.53	2.57
Motherboard 4	482.68	23.1	0.01	0.016	3.76	2.64	1.19	1.67	2.93

**Table 3:** *E*’ values for the involved potential active reaction.

Potential Active Reaction	*E°* [V/HNE]	*E’* [V/HNE]	Thermodynamic Equilibrium Constants $logK_{fox}$
$AuCl_{4}^{-}+3\;e^{-}\to Au+4Cl^{-}$	1.52	1.02	25.42
$AuCl_{2}^{-}+e^{-}\to Au+2Cl^{-}$	1.83	1.25	9.83
$AgCl_{2}^{-}+e^{-}\to Ag+2Cl^{-}$	0.79	0.48	5.25
$CuCl_{2}{}_{aq}+2\;e^{-}\to Cu+2Cl^{-}$	0.34	0.294	1.56
$CuCl_{2}^{-}+e^{-}\to Cu+2Cl^{-}$	0.52	0.21	5.14
$PbCl_{4}^{2-}+2\;e^{-}\to Pb+4Cl^{-}$	−0.126	−0.17	1.6
$PbCl_{6}^{2-}+4\;e^{-}\to Pb+6Cl^{-}$	0.762	0.747	1.05
$SnCl_{4}^{2-}+2\;e^{-}\to Sn+4Cl^{-}$	−0.137	−0.19	1.79
$SnCl_{6}^{2-}+4\;e^{-}\to Sn+6Cl^{-}$	−0.0085	−0.025	1.11
$NiCl_{4}^{2-}+2\;e^{-}\to Ni+4Cl^{-}$	−0.26	−0.28	0.7
$FeCl_{4}^{-}+3\;e^{-}\to Fe+4Cl^{-}$	−0.036	−0.031	−0.09
$FeCl_{4}^{2-}+2e^{-}\to Fe+4Cl^{-}$	−0.44	−0.402	−1.276
$ZnCl_{4}^{2-}+2\;e^{-}\to Zn+4Cl^{-}$	−0.76	−0.77	0.2

**Table 4:** *E’* values for PAR that involve the complexation of both forms of the redox couple.

Potential Active Reaction	*E*^0^ [V/HNE]	*E’* [V/HNE]
$FeCl_{4}^{-}+\;e^{-}\to FeCl_{4}^{2-}$	0.77	0.7
$CuCl_{2}{}_{aq}+e^{-}\to CuCl_{2}^{-}$	0.16	0.37
$PbCl_{6}^{2-}+2e^{-}\to PbCl_{4}^{2-}+2Cl^{-}$	1.65	1.664
$SnCl_{6}^{2-}+2\;e^{-}\to SnCl_{4}^{2-}+2Cl^{-}$	0.154	0.14

**Table 5:** The values of redox equilibrium constants calculated for the leaching reactions.

Redox Reaction	Redox Equilibrium Constant
$3\;FeCl_{4}^{-}+Au+4Cl^{-}⇌\;AuCl_{4}^{-}+3\;FeCl_{4}^{2-}$	$5.35\times 10^{-17}$
$FeCl_{4}^{-}+Au+2Cl^{-}\;⇌\;AuCl_{2}^{-}+\;FeCl_{4}^{2-}$	$4.76\times 10^{-10}$
$FeCl_{4}^{-}+Ag+2Cl^{-}\;⇌\;AgCl_{2}^{-}+\;FeCl_{4}^{2-}$	$5.35\times 10^{3}$
$FeCl_{4}^{-}+Cu+2Cl^{-}\;⇌\;CuCl_{2}^{-}+\;FeCl_{4}^{2-}$	$2.01\times 10^{8}$
$FeCl_{4}^{-}+CuCl_{2}^{-}\;⇌\;CuCl_{2}{}_{aq}+\;FeCl_{4}^{2-}$	$3.91\times 10^{5}$
$2\;FeCl_{4}^{-}+Cu+2Cl^{-}\;⇌\;CuCl_{2}{}_{aq}+2\;\;FeCl_{4}^{2-}$	$5.79\times 10^{13}$
$2\;FeCl_{4}^{-}+Sn+4Cl^{-}\;⇌\;SnCl_{4}^{2-}+2\;FeCl_{4}^{2-}$	$1.47\times 10^{30}$
$2\;FeCl_{4}^{-}+SnCl_{4}^{2-}+2Cl^{-}⇌\;SnCl_{6}^{2-}+2\;FeCl_{4}^{2-}$	$9.61\times 10^{18}$
$4\;FeCl_{4}^{-}+Sn+6Cl^{-}\;⇌\;SnCl_{6}^{2-}+4\;FeCl_{4}^{2-}$	$1.42\times 10^{49}$
$2\;FeCl_{4}^{-}+Pb+4Cl^{-}\;⇌\;PbCl_{4}^{2-}+2\;FeCl_{4}^{2-}$	$3.1\times 10^{29}$
$2\;FeCl_{4}^{-}+PbCl_{4}^{2-}\;⇌\;PbCl_{6}^{2-}+2\;FeCl_{4}^{2-}$	$2.09\times 10^{-33}$
$4\;FeCl_{4}^{-}+Pb+6Cl^{-}\;⇌\;PbCl_{6}^{2-}+4\;FeCl_{4}^{2-}$	$6.5\times 10^{-4}$
$2\;FeCl_{4}^{-}+Ni+4Cl^{-}\;⇌\;NiCl_{4}^{2-}+2\;FeCl_{4}^{2-}$	$1.66\times 10^{33}$
$2\;FeCl_{4}^{-}+Fe+4Cl^{-}\;⇌\;3\;FeCl_{4}^{2-}$	$2.2\times 10^{37}$
$2\;FeCl_{4}^{-}+Zn+4Cl^{-}\;⇌\;ZnCl_{4}^{2-}+2\;FeCl_{4}^{2-}$	$3.1\times 1^{49}$

**Table 6:** Material fractions resulted from the dismantling process.

Plastics	Boards	Chips and Small EC	Sludge	Solution
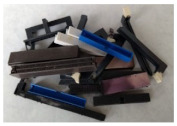	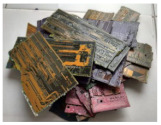	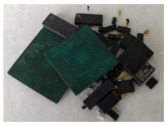	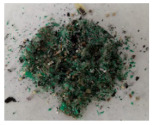	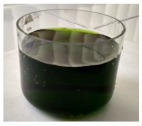

**Table 7:** The metallic composition of the different fractions obtained in the dismantling process.

Sample	Type of Fractions	Total Mass of Fractions	Metallic Composition, %							
			Cu	Au	Ag	Sn	Pb	Ni	Zn	Fe
**Motherboard 1**	Plastics	147.6	-	-	-	-	-	-	-	-
	Boards	174.46	17.2	-	-	-	-	-	-	-
	Chips and small EC	29.21	8.3	0.05	0.1	0.75	0.15	0.75	0.15	1.64
	Sludge	9.73	3.65	0.37	0.05	28.72	36.7	1.09	0.02	0.4
	Dissolved metals	98.14	52.16	0	0.05	15.27	7.12	4.86	7.95	12.62
**Motherboard 2**	Plastics	166	-	-	-	-	-	-	-	-
	Boards	172.2	23	-	-	-	-	-	-	-
	Chips and small EC	35.05	8.4	0.06	0.1	0.55	0.15	0.83	0.13	1.29
	Sludge	5.2	3.1	0.44	0.06	29.53	39.1	1.05	0.022	0.51
	Dissolved metals	110	48.20	0	0.03	16.63	7.70	5.33	8.48	12.93
**Motherboard 3**	Plastics	147.9	-	-	-	-	-	-	-	-
	Boards	170.2	23.5	-	-	-	-	-	-	-
	Chips and small EC	20.8	8.7	0.03	0.1	0.68	0.13	0.91	0.18	1.51
	Sludge	8.1	3.65	0.64	0.05	26.83	34.8	1.02	0.025	0.47
	Dissolved metals	97.06	57.73	0	0.07	13.67	8.92	4.57	6.96	11.40
**Motherboard 4**	Plastics	145.8	-	-	-	-	-	-	-	-
	Boards	141.55	20.2	-	-	-	-	-	-	-
	Chips and small EC	57.25	8.1	0.05	0.1	0.61	0.16	0.81	0.15	1.37
	Sludge	2.65	3.65	0.74	0.05	27.52	38.1	1.12	0.02	0.44
	Dissolved metals	135.43	57.72	0	0.01	12.60	8.60	3.88	5.89	9.85

**Table 8:** Metallic composition (wt.%) of WPCBs feed into the recycling plant.

Cu	Au	Ag	Sn	Pb	Ni	Zn	Fe
20.77	0.011	0.017	3.83	2.42	1.16	1.71	2.84

**Table 9:** Model assumptions and input data [31,32,33,38].

Unit	Parameters
Leaching reactor	Temperature: 25 °C
	Residence time 24 h
	0.3 M FeCl_3_ in 0.5 M HCl
Electrochemical reactor	*r_e_* = 90.12%
	*r_c_* = 72.69%
	*W_c_* = 1.59 kWh/kg
Processed WPCBs	100 kg/h
Pump efficiency	90%
Heat exchanger Δ*T_min._*	10 °C
Heat exchanger pressure drop	1–3%

**Table 10:** Overall mass balance of the scaled-up dismantling process.

**Input**		**Output**	
**Component**	**kg/h**	**Component**	**kg/h**
WPCBs	100	Copper deposit	18.7
Leaching solution	623.4	Sludge	3.7
		Plastic	32.4
		Epoxy resin	14.5
		Fiber glass	15.1
		Depleted solution	634
**TOTAL**	**723.4**	**TOTAL**	**723.4**

**Table 11:** Overall energy balance of the scaled-up dismantling process.

Process Equipment		Energy, (MJ/h)	
Type	ID	Added	Extracted
Crusher	3, 9	0.212	-
Reactor	1	2.08	211.545
Reactor	11	1.07	45.407
Separators	6, 8, 12	25.617	-
Electrochemical Reactor	15	144.17	-
Pumps	5, 10, 13, 18, 19	1.398	-
TOTAL:		174.5470	256.952

## Data Availability

Data sharing is not applicable to this article.
